# Validation and refinement of a *RUNX1* mutation-associated gene expression signature in blast crisis chronic myeloid leukemia

**DOI:** 10.1038/s41375-022-01508-1

**Published:** 2022-02-04

**Authors:** Kian Leong Lee, Tun Kiat Ko, Nicole Y. L. Saw, Asif Javed, Axel M. Hillmer, Charles Chuah, Vaidehi Krishnan, S. Tiong Ong

**Affiliations:** 1 Cancer & Stem Cell Biology Signature Research Programme, Duke-NUS Medical School, Singapore, Singapore; 2 Laboratory of Cancer Epigenome, National Cancer Centre Singapore, Singapore, Singapore; 3 School of Biomedical Sciences, Li Ka Shing Faculty of Medicine, The University of Hong Kong, Hong Kong SAR, China; 4 Cancer Therapeutics & Stratified Oncology, Genome Institute of Singapore, Singapore, Singapore; 5 Institute of Pathology, Faculty of Medicine and University Hospital Cologne, University of Cologne, Cologne, Germany; 6 Department of Haematology, Singapore General Hospital, Singapore, Singapore; 7 Department of Medical Oncology, National Cancer Centre Singapore, Singapore, Singapore; 8 Department of Medicine, Duke University Medical Center, Durham, NC USA

**Keywords:** Chronic myeloid leukaemia, Chronic myeloid leukaemia

## To the Editor:

We note with great interest recent work by Awad et al. [1]. that has identified specific *RUNX1* mutation-related gene expression signatures in blast crisis (BC) chronic myeloid leukemia (CML) patient samples harbouring *RUNX1* mutations. Recurrent mutations in the *RUNX1* tumour suppressor gene are one of the most frequent events in BC CML and are associated with disease progression, poor response and adverse patient outcomes [2]. Mechanistically, RUNX1 mutations that decrease transcriptional activity or exert a dominant negative effect on wild type RUNX1 are thought to block differentiation leading to BC or accelerated phase-like phenotypes in mice [3].

Consequently, new treatment options such as with CD19-CAR T cells, mTOR, BCL2 and VEGFR targeted therapies identified by the authors become important avenues for *RUNX1* mutant BC CML patients. Therefore, it is imperative to accurately diagnose and assess the suitability of BC CML patients for new therapeutic approaches and identify additional molecular vulnerabilities that can improve patient outcomes.

**Fig. 1:**
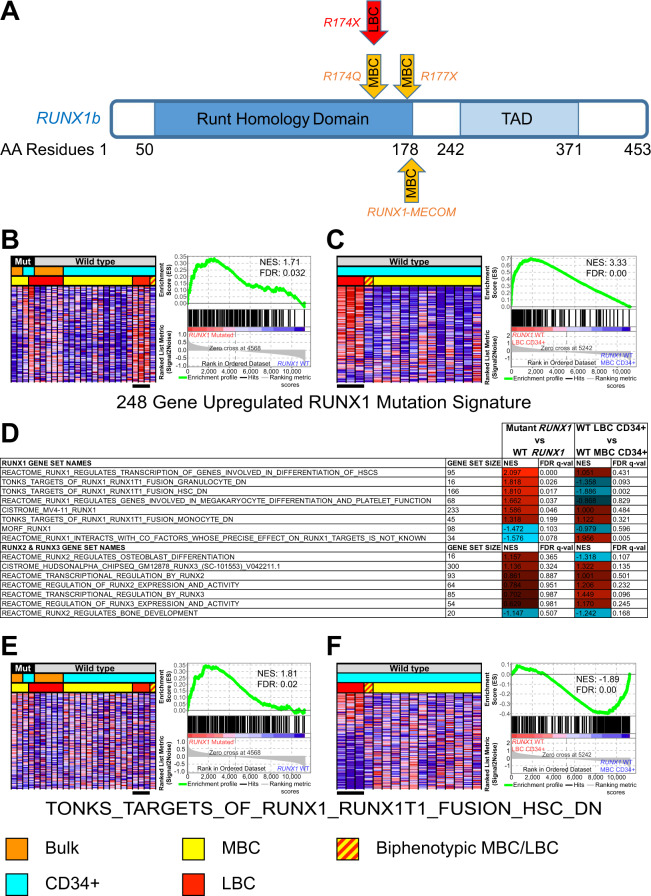
Validation and refinement of *RUNX1* mutational signatures in CML blast crisis patients. **A** Schema of *RUNX1b* gene isoform with mutations found in 3 MBC and 1 LBC CML patients. Horizontal scale indicates amino acid (aa) residue positions. **B** GSEA heatmap in left panel shows expression of genes upregulated in the Awad *RUNX1* mutational signature in *RUNX1* mutant (Mut) compared to wild type patients in red for upregulation and blue for downregulation. Right panel shows enrichment plot with normalised enrichment score (NES) and false discovery rate (FDR). **C** GSEA heatmap in left panel and enrichment plot in right panel shows enrichment of the same gene set in wild type *RUNX1* CD34 + LBC patient gene expression data compared to the equivalent in MBC. **D** Table shows alternative gene sets that can significantly distinguish mutant *RUNX1* blast crisis samples from wild type (WT) in the top half (FDR < 0.25). The same gene sets do not enrich significantly for wild type *RUNX1* CD34 + LBC samples compared to the equivalent in MBC or are depleted. Negative control RUNX2 and RUNX3 gene sets also do not enrich significantly in *RUNX1* mutant compared to wild type samples in the bottom half. **E** GSEA heatmap in left panel shows expression of the TONKS_TARGETS_OF_RUNX1_RUNX1T1_FUSION_HSC_DN gene set [11] with enrichment in *RUNX1* mutant (Mut) compared to wild type patients. Right panel shows enrichment plot with normalised enrichment score (NES) and false discovery rate (FDR). **F** GSEA heatmap in left panel and enrichment plot in right panel shows depletion of the same gene set in wild type *RUNX1* CD34 + LBC compared to the equivalent MBC gene expression data. Black bar indicates 3 CD34 + wild type *RUNX1* LBC samples while yellow and red hatched bar denotes a biphenotypic wild type *RUNX1* sample with characteristics of both LBC and MBC.

In light of this, we examined our own cohort of BC CML patients [4] to identify cases with *RUNX1* mutations and assess some of their molecular features compared to wild type *RUNX1* cases. We recently performed a whole genome and transcriptome-wide study of BC CML patients in bulk bone marrow mononuclear cells and CD34 + leukemic stem cell populations. We identified *RUNX1* aberrations in one lymphoid blast crisis (LBC) and three myeloid blast crisis (MBC) patients. Of these, two MBC cases and oneLBC harbour point mutations in the Runt homology domain that affect DNA binding (Fig. 1A). The remaining MBC patient possesses the t(3;21) translocation resulting in the *RUNX1-MECOM* fusion known to exert a dominant negative effect on normal RUNX1 transcriptional activity [5].

Here, we first confirm that the upregulated genes of the *RUNX1* mutation transcriptional signature (‘Awad’ signature) enrich significantly (Fig. 1B, Supplementary Tables 1 and 2) in our *RUNX1* mutant BC samples in gene set enrichment analysis (GSEA [6]). For downregulated genes of the Awad signature, there were no significant enrichments (Supplementary Tables 1 and 2), and we did not make further use of these genes in this regard as their expression were low or undetectable in our microarrays. Similar results were obtained when we restricted the GSEA analysis to only protein coding genes within the Awad signature (Supplementary Table 3).

Notably, we also found that the upregulated Awad signature was enriched in wild type *RUNX1* CD34 + LBC cases. Three wild type *RUNX1* CD34 + LBC samples showed consistent enrichment for upregulated genes while a fourth sample that was biphenotypic did not show any enrichment (Fig. 1B). To confirm that the Awad signature does indeed significantly co-detect wild type *RUNX1* CD34 + LBC samples and not just *RUNX1* mutant samples, we compared CD34 + LBC against CD34 + MBC where none of the samples carry any *RUNX1* mutations (Fig. 1C and Supplementary Table 2). Indeed, wild type *RUNX1* CD34 + LBC samples were enriched for the upregulated Awad signature genes compared to wild type *RUNX1* CD34 + MBC samples.

Subsequently, we sought to develop more discriminating gene expression signatures and reasoned that direct targets of RUNX1 and genes sets from other studies may be able to detect differences between *RUNX1* mutant versus wild type patients more specifically. We screened and identified 24 gene sets from the CISTROME database [7] curated from RUNX1 ChIP-seq experiments with a focus on human leukemic and hematopoietic studies and 42 gene sets from MSigDB v7.3 [6] for RUNX1-regulated gene expression with RUNX2 and RUNX3 gene sets as negative controls (Supplementary Table 1). We found that some gene sets were able to identify *RUNX1*-mutated BC samples (Fig. 1D and Supplementary Table 2) without significantly detecting wild type *RUNX1* CD34 + LBC samples (Fig. 1E and Supplementary Table 2). To confirm this, we used these gene sets to make a control comparison between wild type *RUNX1* CD34 + LBC and the MBC equivalent, and found no significant concordant enrichments, or there were depletions instead (Fig. 1D, F and Supplementary Table 2).

**Fig. 2:**
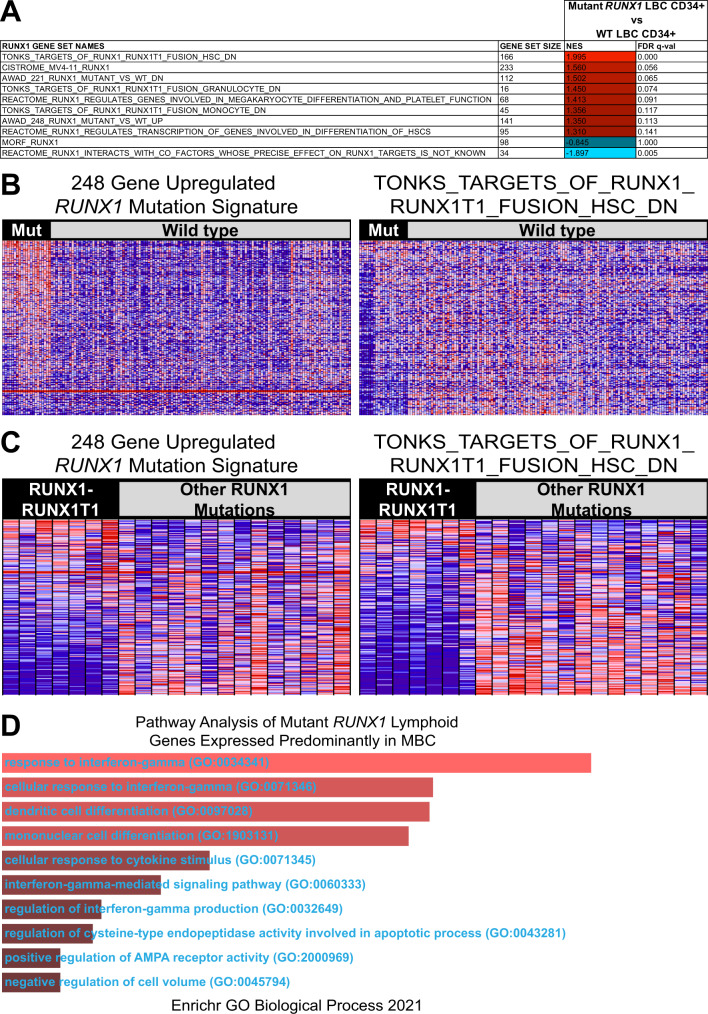
Analysis of Awad and other *RUNX1* mutant signatures in CML LBC and in AML. **A** Table shows GSEA analysis of 1 mutant *RUNX1* CD34 + LBC sample against three wild type *RUNX1* CD34 + LBC samples for the *RUNX1* gene sets indicated. NES scores are denoted in red for upregulation and blue for downregulation. **B** GSEA heatmap shows expression of the upregulated Awad signature gene set in the left panel and TONKS_TARGETS_OF_RUNX1_RUNX1T1_FUSION_HSC_DN gene set in the right panel. 21 *RUNX1* mutant (Mut) TCGA AML patient samples were compared against 130 wild type cases in red for upregulation and blue for downregulation. **C** GSEA heatmaps show the same gene sets with enrichments in 7 *RUNX1-RUNX1T1* TCGA AML patients compared to 14 other patients with *RUNX1* mutations predominantly in the Runt domain. **D** Bar chart shows pathway analysis of mutant *RUNX1-*regulated lymphoid genes aberrantly expressed in predominantly mutant *RUNX1* MBC patient samples. Conserved leading edge *RUNX1* mutant target genes were identified from the Awad cohort and predominantly MBC CML patients in this study (Fig. 1B). 22 *RUNX1* mutation-regulated lymphoid genes were then shortlisted by overlapping with the leading edge of the wild type *RUNX1* LBC versus wild type *RUNX1* MBC comparison (Fig. 1C) and subjected to Enrichr [12] analysis. The top 10 most significant GO terms are inversely ranked by relative p-values.

While the Awad signature co-detects lymphoid genes in wild type *RUNX1* LBC, it may still be able to detect *RUNX1* mutation-related signatures that are distinct from LBC identity per se. Indeed, GSEA analysis of 1 mutant *RUNX1* CD34 + LBC against 3 wild type *RUNX1* CD34 + LBCs showed the Awad and shortlisted *RUNX1* gene sets were significantly enriched (FDR < 0.25) in the mutant *RUNX1* CD34 + LBC sample (except for MORF_RUNX1 which enriched in the correct direction but did not achieve significance [Fig. 2A and Supplementary Table 4]). We conclude that *RUNX1* mutation gene sets can be used to detect *RUNX1* mutation-containing samples in both MBC and LBC, but only when comparing within separate MBC and LBC datasets.

As a further test of the utility of *RUNX1* mutational signatures to detect *RUNX1* targets within leukemic transcriptomes, we next examined a large cohort of 151 AML patients with RNA-Seq data from TCGA [8] (Supplementary Table 5). When tested against mutant and wild type *RUNX1* TCGA AML patients, both the Awad and shortlisted gene sets were also highly enriched in mutant *RUNX1* AML samples (Fig. 2B and Supplementary Table 6). Interestingly, the *RUNX1* mutation gene sets were also able to distinguish AML cases with the *RUNX1-RUNX1T1* fusion from patients with other Runt domain mutations (Fig. 2C and Supplementary Table 7). This observation supports the notion of functional differences between these 2 types of *RUNX1* mutations as recently reported by Kellaway et al. [9].

Next, to understand the potential biological significance of mutant *RUNX1*-regulated lymphoid genes that were aberrantly expressed in mutant *RUNX1* MBC samples, we performed pathway analysis on 22 mutant *RUNX1* lymphoid targets identified from our analysis and found that interferon gamma signalling was highly significant (Fig. 2D). Thus, the lymphoid genes dysregulated by mutant *RUNX1* in MBC may be involved in immune signalling and inflammation, both of which are important hallmarks of BC transformation [4].

Finally, we confirmed that the gene signatures in our *RUNX1* mutant samples are enriched specifically for RUNX1 gene sets as neither RUNX2 nor RUNX3 datasets enrich significantly in *RUNX1* mutant compared to wild type (Fig. 1D and Supplementary Table 2). Therefore, in addition to the Awad signature, the RUNX1 gene sets we identified can be used for more targeted studies of *RUNX1* mutant signatures in CML and AML samples. These refinements may lead to better understanding of the molecular underpinnings of BC CML cases at risk of progressing or becoming resistant to TKI therapies, and also for mutated *RUNX1* AML cases that have poor outcomes. Moreover, these patients may be amenable to new treatments specific to the *RUNX1* mutational context such as through the use of BET inhibitors or proteolysis targeting chimeras (PROTACs) [10].

## Supplementary information

Supplementary Tables 1 to 7
